# Discharge Readiness and Patient Experience in the Hospital-to-Home Transition After Digestive Surgery

**DOI:** 10.3390/jcm15176775

**Published:** 2026-08-31

**Authors:** Fernando Dana, Raquel Sebio-García, Rubèn González-Colom, Beatriz Tena, David Capitán, Dulce Momblan, Eva Rivas, Marina Roger, Paula Isern, María Suárez, Adelaida Zabalegui, Graciela Martínez-Pallí

**Affiliations:** 1 Anesthesiology Department, Hospital Clinic of Barcelona, 08036 Barcelona, Spain; fjdana@clinic.cat (F.D.); gmartin@clinic.cat (G.M.-P.); 2 Universitat de Barcelona (UB), 08007 Barcelona, Spain; 3 Fundació Clínic for Biomedical Research-August Pi I Sunyer Biomedical Research Institute (FCRB-IDIBAPS), University of Barcelona, 08036 Barcelona, Spain; 4 Physical Medicine and Rehabilitation Department, Hospital Clinic of Barcelona, 08036 Barcelona, Spain; 5 Biomedical Research Networking Center on Respiratory Diseases (CIBERES), 28029 Madrid, Spain; 6 Department of General and Digestive Surgery, Hospital Clinic of Barcelona, 08006 Barcelona, Spain; 7 Outcomes Research Consortium, Houston, TX 77030, USA; 8 Department Nutrition and Clinical Dietetics, Hospital Clinic of Barcelona, 08036 Barcelona, Spain; 9 Subdivision of Research and Teaching in Nursing, Hospital Clinic of Barcelona, 08036 Barcelona, Spain

**Keywords:** patient-reported experience measures (PREMs), patient-reported outcome measures (PROMs), QoR-15, enhanced recovery after surgery, digestive surgery, postoperative recovery, discharge readiness, emergency department visits

## Abstract

**Background/Objectives**: Complete postoperative recovery increasingly occurs after hospital discharge, particularly in the context of enhanced recovery after surgery (ERAS) programmes. However, patient-reported experience during the transition from hospital to home after digestive surgery remains poorly characterized. This study aimed to evaluate patient-reported experience and to explore its associations with perceived quality of recovery at discharge, surgical complexity, postoperative complications, and unplanned healthcare utilization. **Methods**: This study analyzed a prospective observational cohort of 361 adult patients undergoing elective digestive surgery at a tertiary care center. Patient-reported experience was assessed using an ad hoc structured questionnaire administered at discharge and 30 days later, covering discharge readiness, information, expectations, and support needs. Quality of recovery was assessed using the QoR-15 at discharge. **Results**: At discharge, 71% of patients reported feeling ready for discharge, whereas 41% agreed that they would have needed a longer hospital stay. At 30 days, 87% reported sufficient home-care information, 69% reported that recovery had met their expectations, 35% perceived discharge as premature, and 23% reported need to contact the hospital. Several ST-PREM domains assessed at discharge or during 30-day follow-up were associated with QoR-15 scores at discharge, with differences exceeding the 6-point minimal clinically important difference (MCID). No consistent differences in patient-reported experience were observed according to surgical complexity. Patients with postoperative complications or emergency department attendance reported less favorable recovery-related experience domains. **Conclusions**: Most patients reported favorable experiences during the hospital-to-home transition. However, a clinically relevant subgroup reported uncertainty, perceived premature discharge, or need for post-discharge support. Combining patient-reported experience measures with QoR-15 may help provide a more comprehensive evaluation of early postoperative recovery.

## 1. Introduction

Postoperative recovery is a dynamic and multidimensional process that extends well beyond the moment of hospital discharge. Although discharge is commonly used as a surrogate marker of favourable clinical evolution and healthcare efficiency, it does not necessarily reflect the patient’s actual readiness to navigate the postoperative period at home or their ability to safely and independently resume daily activities [1,2,3]. In general surgery, the ongoing reduction in hospital length of stay driven largely by the implementation of enhanced recovery after surgery (ERAS) programmes [4,5] has led to improvements in clinical outcomes and greater efficiency in healthcare resource use [6,7]. However, this paradigm shift has also transferred a substantial portion of the recovery process from the hospital setting to the patient’s home, increasing the importance of patients’ readiness for discharge and their capacity to cope with postoperative demands outside the hospital [3,8,9].

In this context, the hospital-to-home transition represents a particularly vulnerable period for surgical patients [10,11,12,13]. Persistent pain, fatigue, functional limitations and difficulties in managing care at home may generate uncertainty and increase reliance on caregivers and the need for unplanned consultations, emergency department visits, or hospital readmissions [14,15,16]. Despite this, traditional discharge criteria continue to rely predominantly on clinical parameters, without systematically incorporating the patient’s subjective perception of their readiness for discharge or their experience during hospitalization [17].

In recent years, the Value-Based Health Care (VBHC) framework has promoted a more patient-centred approach [18,19], focusing on outcomes that matter most to patients and integrating not only traditional clinical variables but also measures directly reported by patients themselves [20,21]. Within this framework, Patient-Reported Experience Measures (PREMs) enable the assessment of key aspects of the care process, including communication with healthcare professionals, quality of information received, readiness for discharge, and the overall recovery experience [22,23]. These measures provide complementary insights to traditional clinical outcomes and facilitate the identification of unmet needs during the postoperative period [3,16,24].

Emerging evidence from general post-discharge populations and elective surgical cohorts suggests that patient-reported experience during the hospital-to-home transition is associated with patient satisfaction, patient-reported outcomes, and subsequent healthcare use [10,22]. However, the available evidence in the field of digestive surgery remains scarce.

Although validated instruments exist to assess postoperative recovery, health-related quality of life, and selected aspects of care included in quality-of-life or patient-experience questionnaires [25,26,27,28,29,30,31], the specific patient experience during the hospital-to-home transition after surgery remains less well characterized [17].

Therefore, the primary objective of this study was to characterize, using PREMs assessed at hospital discharge and 30 days later, how patients undergoing elective digestive surgery perceive the hospital-to-home transition, including readiness for discharge, adequacy of information received, fulfilment of recovery expectations, and perceived need for postoperative support. As exploratory secondary objectives, we assessed whether patient-reported experience was associated with perceived quality of recovery measured using the QoR-15 at hospital discharge, surgical complexity according to the NICE classification, postoperative complications, and emergency department attendance within 30 days after discharge.

## 2. Materials and Methods

### 2.1. Study Design

This study was a secondary analysis of a prospective, single-centre observational cohort conducted at Hospital Clínic de Barcelona, a tertiary university hospital, between February 2024 and February 2025. The present analysis was based on prospectively collected patient-reported experience data obtained as part of the study assessments [32,33,34]. The study protocol was approved by the institutional Ethics Committee (reference HCB/2023/0941), and all participants provided written informed consent prior to enrolment. The manuscript was prepared in accordance with the Strengthening the Reporting of Observational Studies in Epidemiology (STROBE) guidelines for observational studies.

### 2.2. Participants

Consecutive adult patients scheduled for elective digestive surgery requiring at least one overnight hospital stay were included. Patients were enrolled on the day of hospital admission for surgery. Patients whit cognitive impairment, neurological conditions that interfered with questionnaire completion, or language barriers that prevented adequate understanding were excluded. In addition, patients who did not complete the discharge patient-reported experience assessment were excluded from the final analysis. All patients were managed according to standardised institutional ERAS-based perioperative protocols, including homogeneous criteria for hospital discharge.

### 2.3. Outcomes and Procedures

The primary objective of this study was to evaluate patient-reported experience related to the hospital discharge process and postoperative recovery during the first 30 days after elective digestive surgery, using PREMs. Patient-reported experience was assessed at two time points: in person at hospital discharge and, subsequently, 30 after discharge by structured telephone interview. This approach was designed to capture patient experience both during the hospital-to-home transition and after a period of recovery in the home setting, enabling the evaluation of immediate discharge perceptions as well as subsequent experiences over the first postoperative month.

#### 2.3.1. Patient-Reported Experience Assessment

An ad hoc structured questionnaire, referred to as the Surgical Transition PREM (ST-PREM), was developed to evaluate patients’ perceived experience during hospital discharge and the postoperative period. The questionnaire was designed by the research team based on domains previously described in validated instruments assessing patient experience, readiness for discharge, and the hospital-to-home transition, incorporating elements derived from the CAHPS Surgical Care Survey [28], the Patient Experience Questionnaire (PEQ) [29], the Readiness for Hospital Discharge Scale [30], and the Instrument for Assessing Patient Experience of Chronic Illness Care (IEXPAC) [31]. The questionnaire domains and items were developed to capture key aspects of the hospital-to-home transition after surgery, including discharge readiness, adequacy of information received, fulfilment of expectations, and postoperative support needs. Item selection was based on the previous literature on patient experience and discharge preparedness and was reviewed by the multidisciplinary clinical team involved in perioperative care to ensure clinical relevance and content adequacy. Given the absence of a specific instrument focused on patient-reported experience during the transition from hospital to home after digestive surgery, a context-specific questionnaire was developed to explore this care process. Customised PREM questionnaires and adapted patient-experience domains are commonly used in PREM research, particularly when context-specific aspects of care are not fully captured by existing standardised instruments [35]. The ST-PREM was not designed as a psychometrically validated scale, and no overall score was calculated; rather, each item was analysed individually to describe specific domains of patient-reported experience during the hospital-to-home transition.

The first part of the questionnaire, composed of four domains and administered at the time of hospital discharge, evaluated aspects related to readiness to return home, the quality of information received about postoperative care, the perceived adequacy of hospital stay duration, and the perceived need for prolonged hospitalization. The second part, composed of five domains and administered at 30 days after discharge through a structured telephone interview, explored aspects related to information received regarding postoperative home care, perceived clinical improvement compared with the reason for admission, fulfilment of expectations regarding postoperative recovery, retrospective perception of the discharge process, and the need to contact the hospital for surgery-related concerns. Both questionnaire administrations were conducted using the same structured questionnaire and predefined response options to ensure consistency across participants. Responses were recorded using a four-point Likert scale (“strongly agree,” “partially agree,” “partially disagree,” and “strongly disagree”), with an additional “do not know/no answer” option. In addition, the questionnaire included an optional open-ended question aimed at collecting suggestions for improvement related to the care process and hospital-to-home transition.

The instrument was conceived as a longitudinal PREM, administered at two different time points during the care process: at hospital discharge and 30 days after discharge.

In addition, we explored the association between ST-PREM responses and perceived quality of recovery at hospital discharge using the Quality of Recovery-15 (QoR-15) questionnaire, a validated patient-reported outcome measure evaluating multiple dimensions of postoperative recovery [36].

#### 2.3.2. Covariates

The following demographic and clinical variables were collected and analysed as covariates: (i) age; (ii) gender; (iii) Body Mass Index (BMI); (iv) American Society of Anaesthesiologists (ASA) physical status classification [37]; (v) frailty assessed using the Canadian Study of Health and Aging Clinical Frailty Scale [38]; (vi) neoadjuvant treatment; (vii) type of surgery (categorized by complexity as minor, intermediate, or major, according to criteria from the National Institute for Health and Care Excellence (NICE)) [39]; (viii) surgical approach (open, laparoscopic, or robotic); and (ix) duration of surgery (in minutes).

Additionally, postoperative clinical variables and healthcare utilisation measures were collected: (i) admission to the intensive care unit (ICU); (ii) ICU length of stay (LOS); (iii) total hospital LOS; (iv) number and severity of postoperative complications classified according to the Clavien–Dindo classification [40] and summarised using the Comprehensive Complication Index (CCI) [41]; (v) emergency department visits within 30 days after discharge; (vi) hospital readmissions within 30 days after discharge; and (vii) postoperative reinterventions within 30 days after discharge.

### 2.4. Sample Size and Statistical Analysis

#### 2.4.1. Sample Size Considerations

This study was a secondary analysis of a previously described prospective cohort. No formal a priori sample size calculation was performed for the present secondary analysis. The final analytic cohort was determined by the number of eligible patients with available patient-reported experience data. The sample size was considered adequate to support the planned descriptive analyses and exploratory comparisons. For estimation of PREMs, the final sample of 361 patients provides a margin of error of approximately ±5 percentage points at the 95% confidence level for proportions around 50%, supporting adequate precision for descriptive estimates. Exploratory comparisons across clinically relevant subgroups were interpreted cautiously and were intended to identify clinically meaningful patterns rather than to provide confirmatory evidence.

#### 2.4.2. Statistical Considerations

Descriptive analyses were conducted for demographic, clinical, surgical, and postoperative variables, as well as for PREMs. Continuous variables were reported as mean and standard deviation (SD) when normally distributed, or as median and interquartile range (IQR) when skewed. Categorical variables were summarized as absolute frequencies and percentages.

Normality of continuous variables was assessed using the Shapiro–Wilk test. Depending on the distribution, parametric tests (Student’s *t*-test) or non-parametric tests (Mann–Whitney U test) were used for comparisons between groups. Categorical variables were compared using the Chi-squared test or Fisher’s exact test, as appropriate.

For comparative analyses, Likert-scale responses were dichotomized into agreement (“strongly agree” and “partially agree”) and disagreement (“partially disagree” and “strongly disagree”) categories to facilitate interpretation and improve the stability of between-group comparisons. Items were analysed in their original direction and were not reverse-coded. Therefore, agreement responses should be interpreted according to the wording of each item: for positively worded items, agreement reflected a favourable experience, whereas for negatively worded items, agreement reflected perceived need for prolonged hospitalization, premature discharge, or need to contact the hospital after discharge. No overall ST-PREM score was calculated. “Do not know/no answer” responses and missing data were excluded from item-specific inferential analyses. Exploratory comparisons of PREMs were performed according to QoR-15 scores at hospital discharge, surgical complexity, occurrence of in-hospital postoperative complications, and emergency department attendance within 30 days after discharge, using appropriate statistical tests (chi-square or Fisher’s exact tests for categorical variables, and Welch’s *t*-tests, Student’s *t*-tests, or Mann–Whitney U tests for continuous variables, as appropriate; mean differences were reported with 95% confidence intervals). Associations between ST-PREM responses and clinical and postoperative variables were also explored using appropriate univariate analyses. All tests were two-sided, and a *p*-value < 0.05 were considered nominally statistically significant. Given the exploratory nature of the analyses, no formal adjustment for multiple comparisons was applied. Therefore, *p*-values should be interpreted as exploratory and hypothesis-generating rather than confirmatory.

As an additional exploratory analysis, multivariable logistic regression models were fitted to assess factors associated with agreement responses for each ST-PREM item. For each item, responses were dichotomized into agreement and disagreement categories, while “do not know/no answer” responses and missing data were excluded from item-specific models. Models were adjusted for age, sex, QoR-15 score at hospital discharge, ASA physical status, frailty status according to the CSHA Clinical Frailty Scale, surgical approach, surgical complexity according to the NICE classification, length of hospital stay, and in-hospital postoperative complications. Results are reported as adjusted odds ratios (ORs) with 95% confidence intervals (CIs).

All analyses were performed using IBM SPSS Statistics for Windows, version 26.0 (IBM Corp., Armonk, NY, USA), and R software 4.1.0 (R Foundation for Statistical Computing, Vienna, Austria). Analyses were conducted using complete-case data.

## 3. Results

### 3.1. Study Population

**Figure 1:**
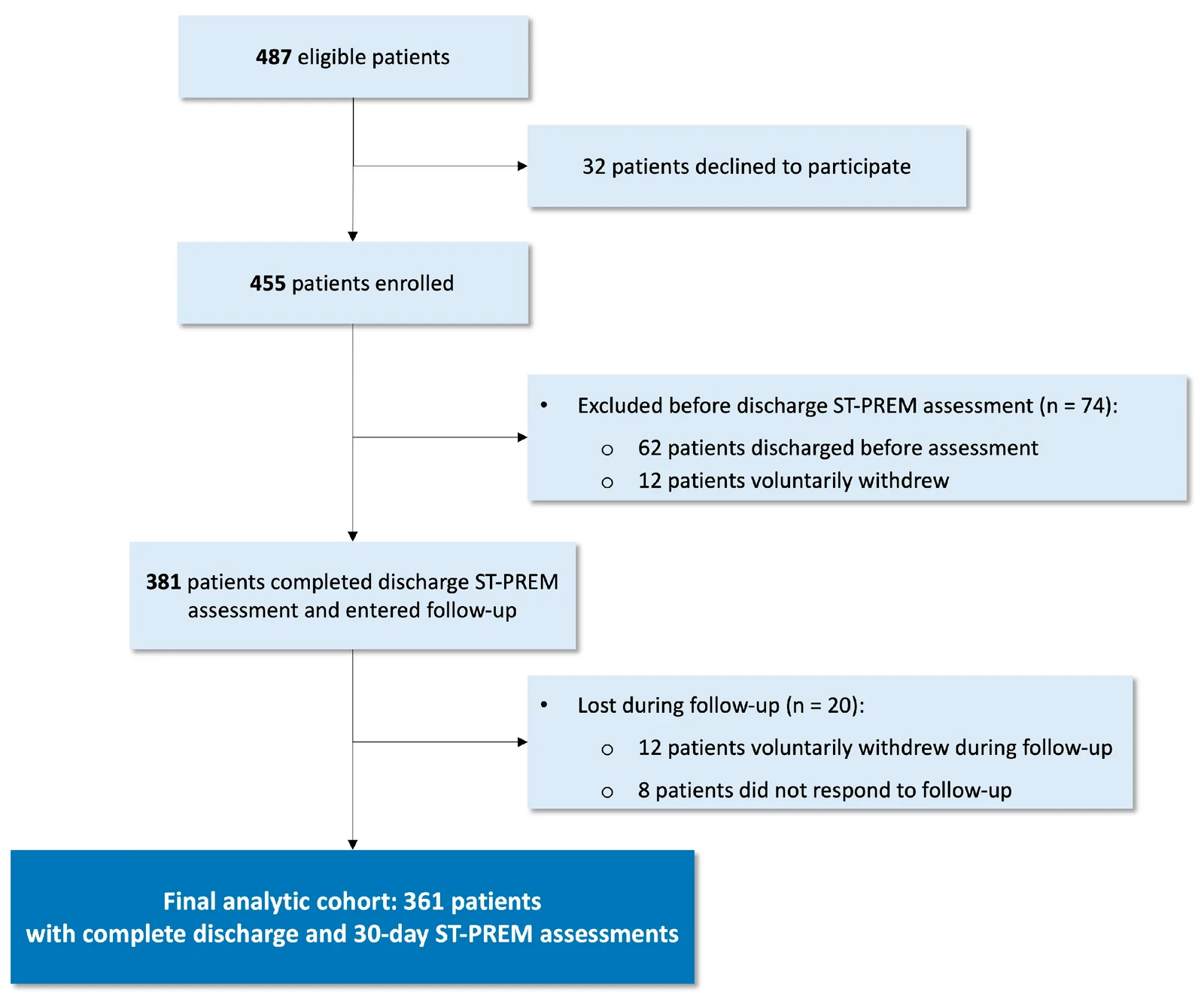
Flowchart of patient selection and completion of ST-PREM assessments.

During the study period, 455 patients provided informed consent before surgery and were enrolled in the study. The first ST-PREM assessment was scheduled at hospital discharge. Seventy-four patients did not complete this assessment: 62 were discharged before it could be administered because of operational and timing-related reasons, and 12 withdrew from the study before hospital discharge. As a result, no discharge ST-PREM data were available for these patients, and they did not enter the 30-day follow-up phase. The remaining 381 patients completed the discharge ST-PREM assessment and entered follow-up. During follow-up, 20 patients did not complete the 30-day assessment: 12 withdrew from the study and 8 did not respond to the telephone follow-up. No postoperative mortality occurred within 30 days after discharge. The final analytic cohort included of 361 patients (Figure 1).

The mean age of the cohort was 65 ± 14 years, and the sex distribution was balanced. Approximately half of the patients were classified as ASA III–IV (49%), and 33% were classified as frail according to the Clinical Frailty Scale. Most procedures were classified as major surgeries (75%), according to the NICE classification, with approximately two-thirds performed using a laparoscopic or robotic approach. The mean duration of surgery was 212 ± 144 min. The full demographic, surgical, and postoperative characteristics of the cohort are presented in Table 1.

### 3.2. Patient-Reported Experience at Discharge (T0) and 30-Day Follow-Up (T30)

Patient-reported experience at both time points is shown in Table 2. At discharge, 71% of patients reported ready for discharge, while 41% agreed that they would have needed a longer hospital stay. In addition, 78% reported having received sufficient information about how they might feel after surgery. At 30 days after discharge, 88% reported having received sufficient information about postoperative home care, 67% perceived an improvement in their health status, and 70% reported that their recovery had met their expectations. Approximately one-third of patients felt that discharge had been premature, and 23% reported needing to contact the hospital because of surgery-related concerns.

### 3.3. Patient-Reported Experience Measures According to Perceived Quality of Recovery and Postoperative Clinical Course

In exploratory comparisons, favourable responses to ST-PREM domains assessed at discharge or during 30-day follow-up were associated with higher QoR-15 scores at hospital discharge (Table 3). The greatest differences were observed for agreement with being ready for discharge (115.1 vs. 106.2; *p* < 0.001) and for fulfilment of recovery expectations (115.2 vs. 105.3; *p* < 0.001). Differences were also observed for information received about postoperative recovery, consistency between hospital stay duration and preoperative information, postoperative home care information, and perceived improvement in health status. Conversely, agreement with the perception that discharge had been premature was associated with lower QoR-15 scores (107.9 vs. 114.8; *p* < 0.001). No nominally significant differences were observed according to perceived need for prolonged hospitalization or need to contact the hospital after discharge.

Exploratory comparisons according to surgical complexity, in-hospital postoperative complications, and emergency department attendance within 30 days after discharge are shown in Table 4. No consistent differences in patient-reported experience were observed according to surgical complexity as defined by the NICE classification across most questionnaire domains. A nominal difference was observed for the perception of having received enough information about how patient might feel after surgery: agreement responses were more frequent among patients undergoing major surgery than among those undergoing minor or intermediate procedures (81% vs. 67% and 71%, respectively; *p* = 0.043). In-hospital postoperative complications were not associated with differences in most patient-reported experience domains. However, as expected, patients with complications reported lower perceived health improvement (62% vs. 77%; *p* = 0.003) and lower fulfilment of recovery expectations (62% vs. 80%; *p* < 0.001). Patients with an emergency department visit within the first 30 days after discharge tended to report lower fulfilment of recovery expectations (60% vs. 75%; *p* = 0.015), a higher perception that discharge had been premature (53% vs. 31%; *p* < 0.001), and a greater need to contact the hospital because of surgery-related concerns (55% vs. 15%; *p* < 0.001). No other nominally significant differences were observed in the remaining domains evaluated.

As an additional exploratory adjusted analysis, multivariable logistic regression models were fitted for agreement responses to each ST-PREM item (Supplementary Table S1). QoR-15 score at hospital discharge showed the most consistent association with ST-PREM agreement responses. After adjustment for age, sex, ASA physical status, frailty status, surgical approach, surgical complexity according to the NICE classification, length of hospital stay, and in-hospital postoperative complications, each 6-point increase in QoR-15 score was associated with higher odds of reporting favourable perceptions across several domains, with adjusted ORs ranging from 1.15 to 1.24. Conversely, higher QoR-15 scores were associated with lower odds of perceiving discharge as premature (adjusted OR 0.85 per 6-point increase; *p* < 0.001). In addition, major surgery was associated with higher odds of reporting sufficient information about how patients might feel after surgery (adjusted OR 2.04; *p* = 0.027), whereas an open surgical approach and in-hospital postoperative complications were associated with lower odds of reporting that recovery had met expectations (adjusted OR 0.56; *p* = 0.033, and adjusted OR 0.47; *p* = 0.008, respectively). The association involving surgical complexity should be interpreted cautiously given the imbalance between NICE surgical complexity groups. Frailty was associated with higher odds of perceiving discharge as premature (adjusted OR 1.80; *p* = 0.036), and longer hospital stay was associated with higher odds of needing to contact the hospital after discharge (adjusted OR 1.04 per additional day; *p* = 0.011). Age, sex, and ASA physical status did not show consistent associations with ST-PREM responses.

## 4. Discussion

This study evaluated patient-reported experience during the transition from hospital to home after elective digestive surgery. The main findings were that, within a standardised perioperative care programme, most patients reported a positive perception of the discharge process and subsequent transition home. However, a substantial proportion expressed uncertainty at discharge or perceived the need for a longer hospital stay, and this perception persisted in a smaller but clinically relevant subgroup 30 days later. These perceptions did not show a consistent pattern according to surgical complexity or according to the occurrence of in-hospital postoperative complications, suggesting that perceived discharge experience may not be solely explained by objective clinical severity. Other factors, such as information provision, communication with healthcare professionals, expectation-setting, symptom burden, confidence in self-care, and perceived continuity of care, may also influence how patients experience the transition from hospital to home [22,42]. Furthermore, patients who attended the emergency department after discharge reported lower fulfilment of recovery expectations and a higher perception that discharge had been premature. Finally, several ST-PREM domains assessed at discharge or during 30-day follow-up were associated with QoR-15 scores at hospital discharge, suggesting a close relationship between quality of recovery and patient experience during the hospital-to-home transition.

These findings are clinically relevant in the context of modern perioperative care and Enhanced Recovery After Surgery (ERAS) programmes, where progressive reductions in hospital length of stay have shifted a substantial component of postoperative recovery to the home setting [4,5,8,43,44,45]. In this setting, concerns have emerged regarding whether increasingly early discharge strategies may affect patients’ perception of safety, preparedness, or continuity of care [10,17,30,46,47]. However, our results suggest that, within a structured perioperative care pathway, most patients reported favourable perceptions of the hospital-to-home transition, despite the complexity of the surgical procedures included in the cohort. Nevertheless, the persistence of uncertainty in a clinically relevant subgroup suggests that subjective recovery experience during the transition to home may involve adaptive and experiential dimensions not fully captured by traditional clinical indicators or conventional surgical outcomes [42,48,49].

Although 71% of patients reported feeling prepared for hospital discharge, 41% also considered that a few additional hospital days might have been beneficial. These findings should not be interpreted as contradictory, as both questions explore different dimensions of the discharge process. Perceived readiness for discharge reflects confidence in continuing recovery at home, whereas the perceived benefit of additional hospitalization may reflect caution, a desire for additional support, or uncertainty regarding early postoperative recovery. This interpretation is reinforced by the 30-day assessment, in which only 35% of patients ultimately considered that their discharge had been premature. Taken together, these findings suggest that some patients may feel prepared to return home while, at the same time, perceiving that a slightly longer period of hospital supervision could have provided additional reassurance during the early postoperative recovery period.

### 4.1. Association Between Patient-Reported Experience Measures and Perceived Postoperative Recovery at Discharge

A consistent pattern observed in this study was that more favourable ST-PREM responses were associated with higher QoR-15 scores at hospital discharge. This pattern was observed both for ST-PREM domains assessed at discharge and for selected domains assessed during 30-day follow-up, including fulfilment of recovery expectations and retrospective perception of the discharge process. Across most evaluated domains, patients reporting favourable perceptions regarding discharge preparation, information received, fulfilment of expectations, and postoperative recovery experience showed higher QoR-15 scores compared with patients reporting less favourable perceptions.

Several patient-reported experience domains showed between-group differences in QoR-15 scores exceeding the commonly accepted 6-point minimal clinically important difference for the QoR-15 [50]. The largest differences were observed for readiness for discharge and fulfilment of postoperative recovery expectations, suggesting that differences in patient-reported experience were not only statistically significant but also potentially meaningful from the patient’s perspective, as they may reflect perceptible differences in overall quality of recovery. These findings suggest a close relationship between discharge experience and perceived postoperative recovery.

From a conceptual perspective, these results support the complementary use of PREMs and PROMs within perioperative care. While PREMs primarily explore how patients perceive the care process and healthcare experience, PROMs such as the QoR-15 evaluate perceived health status and functional recovery. Although these constructs are not interchangeable, our findings suggest that they are closely related during the postoperative period and may reflect overlapping aspects of recovery, adaptation, confidence, symptom burden, and informational needs during the transition from hospital to home [22,23,49,51]. These findings are consistent with previous analyses from this cohort, in which lower QoR-15 scores at hospital discharge were associated with unplanned healthcare use and incomplete recovery of patient-identified meaningful activities after surgery [33,34]. The exploratory multivariable analyses were consistent with this interpretation, as QoR-15 score at hospital discharge showed the most consistent association with ST-PREM responses after adjustment for relevant clinical and perioperative variables. However, selected perioperative factors, including surgical approach, in-hospital postoperative complications, frailty, and length of hospital stay, were also associated with specific domains of patient-reported experience, suggesting that both perceived recovery quality and perioperative clinical course may contribute to how patients evaluate the hospital-to-home transition.

These findings are particularly relevant within current patient-centred and value-based healthcare frameworks, where recovery is increasingly understood as a multidimensional process extending beyond traditional surgical outcomes alone [18,19,21]. In this context, the combined use of PREMs and PROMs may provide a more comprehensive evaluation of postoperative recovery, integrating clinical recovery, patient experience, functional recovery, and perceived well-being into a unified assessment framework [22,23]. This approach is consistent with contemporary recommendations to incorporate outcomes that matter to patients into quality evaluation and care improvement strategies, while maintaining the distinction between experience of care and perceived health recovery [21,22,23].

### 4.2. Patient-Reported Experience According to Clinical Course and Postoperative Events

Patient-reported experience did not show a consistent pattern according to surgical complexity as defined by the NICE classification. However, this finding should be interpreted cautiously because the surgical complexity groups were unbalanced, with a predominance of major procedures and relatively small minor and intermediate surgery groups. Despite the greater clinical burden usually associated with major surgery, we did not observe a pattern of poorer reported experience among patients undergoing major procedures across most questionnaire domains. A nominal difference was observed in the perception of having received enough information about how they might feel after surgery, which was higher among patients undergoing major surgery. This pattern may reflect more structured preoperative counselling and perioperative preparation in this group, with clearer setting of expectations regarding postoperative recovery. Conversely, patients undergoing minor or intermediate procedures may receive less intensive preparatory information, although their need for guidance during the transition home may still be substantial.

The occurrence of in-hospital postoperative complications was mainly associated with domains related to perceived recovery rather than with discharge readiness itself. Patients with complications reported lower perceived health improvement and lower fulfilment of recovery expectations, whereas no clear differences were observed in readiness for discharge, perceived need for prolonged hospitalization, or retrospective perception of premature discharge. These findings suggest that complications may primarily influence how patients interpret their recovery trajectory and whether recovery evolves as expected, rather than the immediate perception of discharge preparedness.

This interpretation is consistent with previous studies showing that postoperative complications are associated with poorer patient-reported outcomes, delayed functional recovery, and reduced quality of life after surgery [52,53]. Complications may influence dimensions of recovery not fully captured by traditional surgical metrics, including symptom burden, fatigue, emotional well-being, and return to normal daily functioning [16,20].

In contrast to surgical complexity and in-hospital complications, emergency department attendance after discharge was associated with a less favourable retrospective experience [14,15,17,47]. Patients who attended the emergency department reported lower fulfilment of recovery expectations and a higher perception that discharge had been premature, suggesting that events occurring after discharge may substantially shape patients’ evaluation of the hospital-to-home transition. This finding is consistent with previous work linking discharge readiness and care transition quality with return to hospital or unplanned healthcare use [14,15,17,47].

### 4.3. Clinical Implications and Value-Based Healthcare Perspective

Our findings suggest that PREMs may provide clinically relevant information regarding the transition from hospital to home that is not fully captured by conventional surgical outcomes alone. Although most patients reported favourable experiences across the evaluated domains, a clinically relevant subgroup expressed uncertainty regarding discharge readiness, perceived need for longer hospitalization, or concern that discharge had been premature. Identifying this subgroup is important, as these perceptions may reflect unmet informational, emotional, or supportive needs during the early recovery period.

From a practical perspective, systematic assessment of discharge experience and postoperative recovery perceptions could help guide more individualised discharge preparation and post-discharge support. This may be particularly relevant for patients expressing uncertainty at discharge, those experiencing postoperative complications, or those requiring unplanned emergency department care after discharge. In this context, perioperative nursing, reinforced patient education, and structured follow-up strategies may play an important role in strengthening continuity of care and supporting safer transitions from hospital to home [2,10,43,48,49].

These findings are aligned with current patient-centred and value-based healthcare models, where evaluation of surgical quality increasingly incorporates not only clinical outcomes but also patient experience and perceived recovery [18,19,21,23].

### 4.4. Limitations

This study has several limitations that should be considered when interpreting the findings. First, it was conducted in a single tertiary care centre, which may limit the generalisability of the results. In addition, exclusion of patients who did not complete the discharge PREM assessment or 30-day follow-up may have introduced selection bias. In most excluded patients, the discharge ST-PREM assessment, which represented the main patient-reported experience measure of the study, was unavailable, preventing assessment of potential differences between included and excluded patients. Second, patient experience was assessed using an ad hoc structured questionnaire that, although informed by previously described patient experience domains and established instruments, has not undergone formal psychometric validation. Therefore, its reliability, construct validity, responsiveness, and measurement properties have not been established. Furthermore, although the questionnaire content was informed by previously described domains and established patient experience instruments, future studies should formally evaluate its psychometric properties before it can be considered a validated measure of patient-reported experience. However, currently available instruments are limited in their ability to comprehensively capture the multidimensional nature of patient experience during the hospital-to-home transition, which supports the use of a context-specific tool in this study. Third, the observational design precludes establishing causal relationships between patient-reported experience and postoperative recovery perceptions. In addition, the cohort included a heterogeneous range of surgical procedures with varying levels of complexity, which may influence recovery perceptions and expectations. However, all patients were managed within the same institutional perioperative care pathways, which may have reduced variability in discharge practices and postoperative follow-up. Furthermore, responses were dichotomised for comparative analyses in order to facilitate interpretation and improve analytical robustness; however, this approach may have reduced sensitivity to detect more subtle differences in patient perceptions. Finally, some questionnaire domains were assessed at the time of discharge whereas others were evaluated retrospectively at 30 days, which may introduce temporal variability and potential recall bias in patient-reported perceptions. Although the 30-day assessment represents a clinically relevant early postoperative time point, it may be insufficient for some patients undergoing major procedures to fully appraise their recovery trajectory or the adequacy of the hospital-to-home transition. The study was conducted in the context of the Spanish public healthcare system, where universal healthcare coverage and strong family support structures may influence patients’ perceptions of discharge and recovery. Consequently, caution is warranted when extrapolating these findings to healthcare systems and sociocultural contexts that differ substantially from our own.

## 5. Conclusions

Most patients undergoing elective digestive surgery reported favourable perceptions regarding hospital discharge and the transition to home, with no consistent differences observed according to surgical complexity as defined by the NICE classification. Postoperative complications were associated with lower perceived recovery and fulfilment of expectations, whereas emergency department attendance after discharge was associated with a less favourable retrospective perception of the transition home. More favourable postoperative experiences were generally accompanied by higher QoR-15 scores at hospital discharge, suggesting a close relationship between patient experience and perceived postoperative recovery. Together, these findings suggest that the complementary use of PREMs and PROMs may provide a more comprehensive and patient-centred evaluation of perioperative care.

## Figures and Tables

**Table 1:** Demographic, surgical, and postoperative characteristics of the study cohort.

Demographic and Clinical Data	All Patients (*n* = 361)
Age, years	65.4 ± 13.7
Gender	
Women/Men	172 (48)/189 (52)
BMI, kg/m^2^	28.2 ± 7.6
CSHA CFS	
*Grades I to III/Grades IV to VI*	243 (67)/118 (33)
ASA	
*I/II/III/IV*	11 (3)/172 (48)/169 (47)/9 (2)
Neoadjuvant therapy	
Yes/No	63 (17)/298 (83)
Surgical approach	
*Open/Laparoscopy/Robotic*	122 (34)/150 (42)/89 (24)
Surgical complexity according to NICE	
*Minor/Intermediate/Major*	55 (15)/34 (10)/272 (75)
Surgical time, minutes	212.1 ± 143.8
**Postoperative outcomes**	
ICU admission	102 (28)
ICU LOS (days)	2.7 ± 3.7
Patients with in-hospital complications	163 (45)
Total number of complications	0.9 ± 1.3
CCI	10.1 ± 14.0
LOS (days)	6.3 ± 8.6
Post-discharge emergency department visits	74 (20)
Post-discharge readmissions	36 (10)
Post-discharge reinterventions	9 (2)

Values are presented as *n* (%) or mean ± standard deviation (SD), as appropriate. ASA: American Society of Anaesthesiologists physical status classification; BMI: body mass index; CCI: Comprehensive Complication Index; CSHA CFS: Canadian Study of Health and Aging Clinical Frailty Scale; ICU: intensive care unit; LOS: length of stay. ICU LOS was calculated only among patients admitted to the ICU.

**Table 2:** Patient-reported experience measures at hospital discharge and 30 days after discharge.

ST-PREM Item	Strongly Agree	Partially Agree	Partially Disagree	Strongly Disagree	DNK/NA
**Questions at discharge**					
Do you feel ready to be discharged today?	190 (52)	65 (18)	35 (10)	71 (20)	0 (0)
Do you feel that you received enough information about how you might feel after surgery?	216 (60)	64 (17)	40 (11)	39 (11)	2 (1)
Do you think you would need to stay in hospital for a few more days?	119 (33)	30 (8)	8 (2)	199 (55)	5 (2)
Did the length of your hospital stay match the information you received before admission?	173 (48)	65 (18)	18 (5)	97 (27)	8 (2)
**Questions at 30 days after discharge**					
Did you receive enough information about the postoperative care you would need at home?	257 (71)	59 (16)	17 (5)	24 (7)	4 (1)
Do you think your health condition has improved compared with the reason for your hospital admission?	129 (36)	111 (31)	45 (12)	60 (17)	16 (4)
Has your recovery over the past few weeks met your expectations?	143 (39)	108 (30)	40 (11)	60 (17)	10 (3)
Looking back now, 30 days after discharge, do you think your discharge was premature (that you would have needed a longer hospital stay)?	84 (23)	42 (12)	12 (3)	218 (60)	5 (2)
Did you need to contact the hospital because of surgery-related concerns (excluding emergency department visits)?	73 (20)	10 (3)	4 (1)	269 (74)	5 (2)

Values are presented as *n* (%). Items are presented in their original direction; therefore, agreement with negatively worded items reflects agreement with the perception of needing a longer hospital stay, premature discharge, or need to contact the hospital after discharge. ST-PREM: Surgical Transition Patient-Reported Experience Measure, an ad hoc structured questionnaire used to assess patient-reported experience during the hospital-to-home transition after surgery.

**Table 3:** Quality of Recovery-15 questionnaire scores at hospital discharge according to agreement with ST-PREM items.

ST-PREM Item	Agreement Responses	Disagreement Responses	Mean Difference (95% CI)	*p* Value
**Questions at discharge**				
Do you feel ready to be discharged today?	115.1 ± 15.2	106.2 ± 17.5	+8.9 (+5.1 to +12.8)	<0.001
Do you feel that you received enough information about how you might feel after surgery?	114.0 ± 16.0	107.1 ± 16.6	+6.9 (+2.7 to +11.0)	0.001
Do you think you would need to stay in hospital for a few more days?	110.8 ± 16.5	113.7 ± 16.3	−2.9 (−6.4 to +0.6)	0.101
Did the length of your hospital stay match the information you received before admission?	115.1 ± 15.2	106.9 ± 17.1	+8.2 (+4.5 to +11.9)	<0.001
**Questions at 30 days after discharge**				
Did you receive enough information about the postoperative care you would need at home?	113.4 ± 16.4	104.9 ± 14.0	+8.4 (+3.7 to +13.2)	<0.001
Do you think your health condition has improved compared with the reason for your hospital admission?	114.6 ± 15.8	106.9 ± 16.4	+7.7 (+4.0 to +11.5)	<0.001
Has your recovery over the past few weeks met your expectations?	115.2 ± 15.5	105.3 ± 16.1	+9.9 (+6.2 to +13.7)	<0.001
Looking back now, 30 days after discharge, do you think your discharge was premature (that you would have needed a longer hospital stay)?	107.9 ± 17.3	114.8 ± 15.3	−7.0 (−10.6 to −3.3)	<0.001
Did you need to contact the hospital because of surgery-related concerns (excluding emergency department visits)?	111.1 ± 17.9	112.8 ± 15.9	−1.7 (−6.0 to +2.7)	0.445

Values are presented as mean ± standard deviation (SD) and mean difference with 95% confidence interval (CI). Agreement responses were defined as “strongly agree” or “partially agree”, whereas disagreement responses were defined as “partially disagree” or “strongly disagree”. Items are presented in their original direction; therefore, agreement with negatively worded items reflects agreement with the perception of needing a longer hospital stay, premature discharge, or need for hospital contact after discharge. “Do not know/no answer” responses were excluded from item-specific comparative analyses. Between-group comparisons of QoR-15 scores were performed using two-tailed Welch’s *t*-tests. A *p* value < 0.05 was considered nominally statistically significant. CI: confidence interval; ST-PREM: Surgical Transition Patient-Reported Experience Measure.

**Table 4:** Patient-reported experience measures according to surgical complexity, postoperative complications, and emergency department attendance within 30 days after discharge.

ST-PREM Item	Minor Surgery (*n* = 55)	Intermediate Surgery (*n* = 34)	Major Surgery (*n* = 272)	*p* Value	No In-Hospital Postoperative Complications (*n* = 198)	In-Hospital Postoperative Complications (*n* = 163)	*p* Value	No emergency Department Visit (*n* = 287)	Emergency Department Visit (*n* = 74)	*p* Value
**Questions at discharge**										
Do you feel ready to be discharged today?	37 (67)	27 (79)	191 (70)	0.453	141 (71)	114 (70)	0.882	210 (73)	45 (61)	0.053
Do you feel that you received enough information about how you might feel after surgery?	37 (67)	24 (71)	219 (81)	0.043	156 (80)	124 (76)	0.501	226 (79)	54 (73)	0.311
Do you think you would need to stay in hospital for a few more days?	22 (43)	10 (30)	117 (43)	0.369	82 (42)	67 (42)	1.000	118 (42)	31 (42)	1.000
Did the length of your hospital stay match the information you received before admission?	35 (69)	21 (66)	182 (67)	0.960	137 (72)	101 (62)	0.078	193 (69)	45 (61)	0.220
**Questions at 30 days after discharge**										
Did you receive enough information about the postoperative care you would need at home?	47 (89)	30 (88)	239 (89)	0.998	176 (91)	140 (86)	0.208	254 (90)	62 (84)	0.219
Do you think your health condition has improved compared with the reason for your hospital admission?	29 (60)	23 (70)	188 (71)	0.327	141 (77)	99 (62)	0.003	197 (72)	43 (60)	0.058
Has your recovery over the past few weeks met your expectations?	31 (62)	24 (75)	196 (73)	0.265	151 (80)	100 (62)	<0.001	207 (75)	44 (60)	0.015
Looking back now, 30 days after discharge, do you think your discharge was premature (that you would have needed a longer hospital stay)?	21 (40)	8 (24)	97 (36)	0.260	67 (35)	59 (36)	0.796	87 (31)	39 (53)	<0.001
Did you need to contact the hospital because of surgery-related concerns (excluding emergency department visits)?	12 (22)	6 (18)	65 (24)	0.677	40 (21)	43 (27)	0.234	43 (15)	40 (55)	<0.001

Values are presented as agreement responses *n* (%), corresponding to patients reporting “strongly agree” or “partially agree” for each questionnaire item. Items are presented in their original direction; therefore, agreement with negatively worded items reflects agreement with the perception of needing a longer hospital stay, premature discharge, or need to contact the hospital after discharge. “Do not know/no answer” responses and missing data were excluded from item-specific comparative analyses. Surgical complexity was categorised according to the National Institute for Health and Care Excellence (NICE) classification into minor, intermediate, and major surgery. Postoperative complications were defined as any in-hospital complication occurring during the index admission. Emergency department visits refer to unplanned emergency department attendance within 30 days after hospital discharge. Between-group comparisons were performed using chi-square or Fisher’s exact tests according to expected frequencies. A *p* value < 0.05 was considered nominally statistically significant. ST-PREM: Surgical Transition Patient-Reported Experience Measure.

## Data Availability

The datasets generated and analyzed during the current study are not publicly available due to ethical and legal restrictions under applicable data protection regulations. Data may be made available by the corresponding author upon reasonable request and subject to approval by the relevant ethics committee.

## Supplementary Materials

The following supporting information can be downloaded at: https://www.mdpi.com/article/10.3390/jcm15176775/s1, Table S1: Exploratory multivariable logistic regression models for agreement responses to ST-PREM items.

## Abbreviations

The following abbreviations are used in this manuscript:

ASA	American Society of Anaesthesiologists
BMI	Body Mass Index
CAHPS	Consumer Assessment of Healthcare Providers and Systems
CCI	Comprehensive Complication Index
CI	Confidence Interval
CSHA CFS	Canadian Study of Health and Aging Clinical Frailty Scale
DNK/NA	Do not know/no answer
ERAS	Enhanced Recovery After Surgery
ICU	Intensive Care Unit
IEXPAC	Instrument for Assessing Patient Experience of Chronic Illness Care
IQR	Interquartile Range
LOS	Length of Stay
MCID	Minimal Clinically Important Difference
NICE	National Institute for Health and Care Excellence
PEQ	Patient Experience Questionnaire
PREMs	Patient-Reported Experience Measures
PROMs	Patient-Reported Outcome Measures
QoR-15	Quality of Recovery-15 questionnaire
RHDS	Readiness for Hospital Discharge Scale
SD	Standard Deviation
SPSS	Statistical Package for the Social Sciences
ST-PREM	Surgical Transition Patient-Reported Experience Measure
STROBE	Strengthening the Reporting of Observational Studies in Epidemiology
T0	Hospital discharge assessment
T30	30-day follow-up assessment
VBHC	Value-Based Health Care
