# Supplementary material for: Discharge Readiness and Patient Experience in the Hospital-to-Home Transition After Digestive Surgery
Source: J Clin Med. 2026 Aug 31;15(17):6775. doi: 10.3390/jcm15176775 (PMC13565981; doi:10.3390/jcm15176775)
Supplement: Supplementary file 1 [file jcm-15-06775-s001.zip › jcm-4486986-supplementary.pdf]

Supplementary Table S1. Exploratory multivariable logistic regression models for agreement responses to ST-PREM items

ST-PREM item	Agreement responses	QoR-15 at discharge, per 6-point increase	p value	Other adjustment variables with p < 0.05
Questions at discharge				
Do you feel ready to be discharged today?	255 (70.6)	1.23 (1.12–1.35)	< 0.001	No other variables with p < 0.05
Do you feel that you received enough information about how you might feel after surgery?	280 (78.0)	1.15 (1.05–1.26)	0.004	Major surgery according to NICE: 2.04 (1.08–3.86); p = 0.027
Do you think you would need to stay in hospital for a few more days?	149 (41.9)	0.93 (0.86–1.01)	0.104	No other variables with p < 0.05
Did the length of your hospital stay match the information you received before admission?	238 (67.4)	1.19 (1.08–1.30)	< 0.001	No other variables with p < 0.05
Questions at 30 days after discharge				
Did you receive enough information about the postoperative care you would need at home?	316 (88.5)	1.19 (1.06–1.33)	0.004	No other variables with p < 0.05
Do you think your health condition has improved compared with the reason for your hospital admission?	240 (69.6)	1.18 (1.07–1.29)	0.001	No other variables with p < 0.05
Has your recovery over the past few weeks met your expectations?	251 (71.5)	1.24 (1.13–1.37)	0.001	Open surgical approach: 0.56 (0.33–0.95); p = 0.033<br>in-hospital postoperative complications: 0.47 (0.26–0.82); p = 0.008
Looking back now, 30 days after discharge, do you think your discharge was premature (that you would have needed a longer hospital stay)?	126 (35.4)	0.85 (0.78–0.93)	0.001	CSHA CFS IV–VI: 1.80 (1.04–3.11); p = 0.036
Did you need to contact the hospital because of surgery-related concerns (excluding emergency department visits)?	83 (23.3)	0.99 (0.90–1.09)	0.828	Length of hospital stay, per additional day: 1.04 (1.01–1.07); p = 0.011.

Supplementary Table S1. Values are presented as adjusted odds ratios (ORs) with 95% confidence intervals (CIs) and p values. Agreement responses are presented as n (%) of valid item-specific responses. Each row represents a separate multivariable logistic regression model with agreement response as the dependent variable. Agreement responses were defined as "strongly agree" or "partially agree", whereas disagreement responses were defined as "partially disagree" or "strongly disagree". "Do not know/no answer" responses and missing data were excluded from item-specific models. All models were adjusted for age, sex, QoR-15 score at hospital discharge, ASA physical status, frailty status according to the CSHA Clinical Frailty Scale, surgical approach, surgical complexity according to the NICE classification, length of hospital stay, and in-hospital postoperative complications. Age was modelled per 10-year increase, QoR-15 score per 6-point increase, and length of hospital stay per additional day. The "Other adjustment variables with p < 0.05" column presents adjustment variables with p < 0.05. ASA: American Society of Anaesthesiologists; CI: confidence interval; CSHA CFS: Canadian Study of Health and Aging Clinical Frailty Scale; NICE: National Institute for Health and Care Excellence; OR: odds ratio; QoR-15: Quality of Recovery-15 questionnaire.
ST-PREM: Surgical Transition Patient-Reported Experience Measure.
